# Cause-specific neonatal mortality: analysis of 3772 neonatal deaths in Nepal, Bangladesh, Malawi and India

**DOI:** 10.1136/archdischild-2014-307636

**Published:** 2015-05-13

**Authors:** Edward Fottrell, David Osrin, Glyn Alcock, Kishwar Azad, Ujwala Bapat, James Beard, Austin Bondo, Tim Colbourn, Sushmita Das, Carina King, Dharma Manandhar, Sunil Manandhar, Joanna Morrison, Charles Mwansambo, Nirmala Nair, Bejoy Nambiar, Melissa Neuman, Tambosi Phiri, Naomi Saville, Aman Sen, Nadine Seward, Neena Shah Moore, Bhim Prasad Shrestha, Bright Singini, Kirti Man Tumbahangphe, Anthony Costello, Audrey Prost

**Affiliations:** 1 University College London, Institute for Global Health, London, UK; 2 Diabetic Association of Bangladesh, Perinatal Care Project, Dhaka, Bangladesh; 3 Society for Nutrition, Education and Health Action, Mumbai, India; 4 Parent and Child Health Initiative, Lilongwe, Malawi; 5 Mother and Infant Research Activities, Kathmandu, Nepal; 6 Ministry of Health, Government of Malawi, Lilongwe, Malawi; 7 Ekjut, Chakradharpur, India; 8 MaiMwana Project, Mchinji, Malawi

**Keywords:** Neonatology, Mortality, Epidemiology, Measurement, Data Collection

## Abstract

**Objective:**

Understanding the causes of death is key to tackling the burden of three million annual neonatal deaths. Resource-poor settings lack effective vital registration systems for births, deaths and causes of death. We set out to describe cause-specific neonatal mortality in rural areas of Malawi, Bangladesh, Nepal and rural and urban India using verbal autopsy (VA) data.

**Design:**

We prospectively recorded births, neonatal deaths and stillbirths in seven population surveillance sites. VAs were carried out to ascertain cause of death. We applied descriptive epidemiological techniques and the InterVA method to characterise the burden, timing and causes of neonatal mortality at each site.

**Results:**

Analysis included 3772 neonatal deaths and 3256 stillbirths. Between 63% and 82% of neonatal deaths occurred in the first week of life, and males were more likely to die than females. Prematurity, birth asphyxia and infections accounted for most neonatal deaths, but important subnational and regional differences were observed. More than one-third of deaths in urban India were attributed to asphyxia, making it the leading cause of death in this setting.

**Conclusions:**

Population-based VA methods can fill information gaps on the burden and causes of neonatal mortality in resource-poor and data-poor settings. Local data should be used to inform and monitor the implementation of interventions to improve newborn health. High rates of home births demand a particular focus on community interventions to improve hygienic delivery and essential newborn care.

**What is already known on this topic?**

- Globally, there are three million neonatal deaths every year, mostly from prematurity, asphyxia and sepsis.
- Ninety-eight per cent of these deaths occur in low-income countries, where poor mortality data hinders intervention strategies.
- More direct measurement could guide policy and practice at national and subnational levels.

**What this study adds?**

- Population-based verbal autopsy (VA) tools such as InterVA offer a standardised method to directly measure the burden and causes of neonatal mortality in low-income settings.
- Although patterns of neonatal death differ between sites, both early and late neonatal mortality remain unacceptably high.
- Localised, direct measurement reveals important subnational variations in mortality rates and causes, which might be masked by estimation methods at the national level.

## Introduction

Each year, approximately three million children die in the first 28 days after birth, predominantly due to complications of preterm birth, asphyxia and sepsis.^1–3^ With postneonatal mortality declining faster than neonatal mortality,^4^ these deaths account for a growing proportion of under-five deaths. Understanding the numbers and causes of neonatal deaths, as well as gender differences and national and subnational variation, is key to realising the Every Newborn Action Plan and post-Millennium Development Goals of a ‘grand convergence’ in health, with substantial reductions in neonatal and child mortality.^5–8^ Unfortunately, the resource-poor settings that bear the burden of more than 98% of neonatal deaths often lack the effective vital registration systems crucial to understanding mortality and planning services or interventions.

Advances in epidemiological modelling methods have recently been applied to pooled datasets to characterise neonatal mortality.^5^ ^9^ Such estimates are useful at a global level, but have limitations. Broad underlying assumptions, lack of transparency in the data and methods used and restrictions on disaggregating the data limit their relevance at subnational levels, where there can be substantial variation in rates, trends and cause distributions. There is a growing recognition that measurement rather than modelling is needed^10^ ^11^ and frustration at unsatisfactory progress with civil registration systems. Global bodies, including the WHO, call for the application of fit-for-purpose methods for registering deaths and assigning their causes in a consistent, systematic and timely manner.^12–16^

Since 2001, we have implemented community-based surveillance of perinatal events along with verbal autopsies (VA) in rural Nepal, Bangladesh, Malawi and urban and rural India. This has enabled us to prospectively document births, neonatal deaths and their causes within geographical areas covering a total population of approximately 2.4 million. Using these data, we describe the rates, timing and causes of neonatal mortality for 3772 deaths from low-income and middle-income settings, highlighting regional and gender disparities and identifying priorities for public health intervention.

## Methods

### Study populations

We used data gathered between 2001 and 2011 in cluster randomised controlled trials of community mobilisation ‘women's group’ interventions in Bangladesh (Perinatal Care Project (PCP)^17^ ^18^), Malawi (MaiMwana^19^ and MaiKhanda^20^), India (Society for Nutrition, Education and Health Action (SNEHA)^21^ and Ekjut^22^) and Nepal (Makwanpur and Dhanusha^23^). The data represent 118 084 births recorded in seven locations with community-based surveillance of perinatal events. Characteristics of each study population, study timelines and basic neonatal health indicators are summarised in table 1. Given the documented effect of the women's group intervention on neonatal mortality,^24^ only data from counterfactual clusters without women's groups were included in the analysis. Counterfactual clusters were not pure control areas in every setting: in MaiMwana, Malawi, a breastfeeding counselling intervention was implemented and tested in 12 of the 24 counterfactual clusters;^19^ in PCP, Bangladesh, four of the nine clusters received training of traditional birth attendants on resuscitation;^25^ in Dhanusha, Nepal, a community-based neonatal sepsis management intervention was implemented in 9 of the 30 counterfactual clusters.^26^

**Table 1:** Study populations in descending order of neonatal mortality rate

						InterVA disease prevalence settings	
Study site and area description	Cluster definition	Clusters included	Period of mortality surveillance	VA completion rate as proportion of recorded neonatal deaths (reference)	Estimated % institutional births during data collection	Malaria	HIV
*Ekjut—rural India*: three rural districts in Jharkhand (Saraikela Kharsawan, West Singhbhum) and Odisha (Keonjhar)^22^	8–10 villages with residents classified as tribal/scheduled caste or other backward caste: average population 6338	18	Aug 2005–Jul 2008	98%^22^	20%^22^	High	Very low
*SNEHA—urban India*: six municipal wards in Mumbai^21^	Slum area: population ∼1000 residents	24	Oct 2005–Feb 2010	60%^44^	87%^21^	Low	Very low
*Dhanusha—rural Nepal*: district excluding Janakpur municipality^26^	Village development committee: population ∼8000	30	Jun 2006–Apr 2011	70% (51)	26%^63^	Very low	Very low
*Makwanpur—rural Nepal*: village development committee areas in Makwanpur district^23^	Village development committee: population ∼7000	12	Apr 2001–Oct 2008	98%*	2%^23^	Very low	Very low
*(PCP—rural Bangladesh*: nine unions in three rural districts (Bogra, Faridpur and Moulavibazar)^17^ ^18^	Union (lowest administrative unit): population 25 000–30 000	9	Nov 2004–Jul 2011	83%^17^ ^18^	16%–28%^17^ ^18^	Very low	Very low
*MaiMwana—rural Malawi*: Mchinji district^19^ ^29^	Census enumeration area: population ∼3000	24	Jun 2004–Jan 2011	92%*	37%–44%^19^	High	High
*MaiKhanda—rural Malawi*: three districts in central region (Kasungu, Lilongwe and Salima)^20^	Health centre catchment area: population ∼30 000	31 (sample of 4000 from each cluster)	Jun 2007–Dec 2010	86%^30^	50%–67%^20^	High	High

*Estimated from available data.

PCP, Perinatal Care Project; SNEHA, Society for Nutrition, Education and Health Action; VA, verbal autopsies.

### Community-based surveillance and verbal autopsy

Surveillance systems for births and newborn deaths were established in each setting. Their design and implementation have been described elsewhere.^17^ ^20^ ^21^ ^23^ ^26–30^ Field-based key informants recruited in cluster subareas identified births and deaths, which were then verified by trained interviewers. At all sites, stillbirths were distinguished from very early neonatal deaths based on the absence of signs of life (movement, breathing, crying) when the infant was born, according to the mother or other caregivers. In MaiKhanda, Malawi, a simple algorithm was used to classify deaths as stillbirths or neonatal deaths from data pertaining to these reported signs of life.^30^

To ascertain likely causes of neonatal deaths, trained lay interviewers conducted VAs. VA is the process of interviewing a caregiver, relative or witness to ascertain the presence, absence or nature of signs, symptoms and circumstances observed at or around the time of death.^31^ Interviewers used a structured questionnaire to gather VA information, usually from the mother of the deceased child. Questionnaires comprised a series of fixed-response questions, with space to record open-ended comments, but this open-ended information was not included in the current analysis, as previous work has shown that it does not add to biomedical interpretations of cause of death using automated methods.^32^ The questionnaires were similar in all settings (see online supplementary table S1), and interviewers were trained to ask all relevant questions on the VA questionnaire, adhering to predefined skip patterns, regardless of perceived relevance to the specific case. Interviews were conducted after a culturally appropriate mourning period. Only VAs with sex and age at death data were considered to be complete and included in the study. The VA completion rates for each site are summarised in table 1.

### Data management and quality control

Registered vital events identified by incentivised key informants or salaried enumerators were confirmed by interviewer visits to households and, in a subsample of cases, through field supervisor visits. Trends were compared with local public system registration figures. Further quality checks during in-country electronic data entry mandated referral back to the field for correction when errors were detected.^17–23^ ^26–30^

### Interpreting verbal autopsy

VA data were interpreted through InterVA V.4.02 (http://www.interva.net), an automated method for interpreting signs, symptoms and circumstances.^33^ Based on the reported presence or absence of indicators, Bayesian reasoning is applied to calculate the likelihood of 60 possible cause-of-death categories compatible with the International Classification of Diseases V.10. Following procedures outlined in the InterVA user guide,^34^ variables describing specific signs and symptoms were mapped to equivalent InterVA indicators to generate files to be processed through the Bayesian model. For example, any positive responses to questions about intermittent or fast breathing, chest in-drawing, grunting or nasal flaring before death were used to generate a positive response in InterVA's ‘difficulty breathing’ indicator. One member of the research team (EF) reviewed the mapping between each study site's data and the InterVA input file as a quality control measure to check plausibility of symptom frequencies.

InterVA requires population HIV and malaria prevalence to be specified so that the model can account for baseline differences between locations. Previous research has shown that simple descriptions of ‘very low’, ‘low’ or ‘high’ prevalence, corresponding to increasing orders of magnitude, are adequate for this.^35^ Malaria and HIV settings for each site are summarised in table 1.

InterVA reports the probability of up to three of the most likely causes for each death.^33^ We summed the likelihoods of each cause from every individual death to estimate the burden of each at population level. Dividing this estimate by the total number of deaths provided population cause-specific mortality fractions (CSMFs), and dividing by the number of live births at each study site provided estimates of cause-specific mortality rates (CSMRs). In MaiMwana (Malawi) and all Asian sites, it was possible to calculate mortality rates by sex, based on the directly measured number of liveborn males and females. In MaiKhanda (Malawi), however, sex was not recorded for all live births, and estimates of the numbers of liveborn males and females were derived by splitting the total number on the basis of the 2010 Demographic and Health Survey live-birth sex ratio for 2007–2010.^36^

### Ethical considerations

The trials in which the current data were gathered were approved by either the University College London or the Great Ormond Street Hospital Research Ethics Committee in the UK, and by ethical review committees in each setting: the Ethical Review Committee of the Diabetic Association of Bangladesh, the Nepal Health Research Council (with a Memorandum of Understanding with the Government of Nepal Ministry of Health), the Independent Ethics Committee for Research on Human Subjects (Mumbai), an independent ethical committee in Jamshedpur, India, and the Malawi National Health Sciences Research Committee.

## Results

A total of 3772 neonatal deaths with complete age and sex data were included in the analysis. The average time from death to VA interview varied between study sites, with an average of 3 weeks at Ekjut and SNEHA (India), 5–6 weeks at PCP (Bangladesh) and Dhanusha (Nepal), 29 weeks at MaiMwana and 67 weeks at MaiKhanda (Malawi). The relatively long death-to-interview time in MaiKhanda reflects the fact that a large number of VAs were not completed initially, but rather included as part of a verification exercise after the trial ended in May 2011.^30^ Interview dates were not available for Makwanpur (Nepal). The average number of VA indicators per case was 12 for MaiKhanda, 13 for Makwanpur, 14 for Dhanusha, Ekjut and MaiMwana, 15 for SNEHA and 17 for PCP. Numbers of stillbirths and neonatal deaths contributed by each study site are presented in table 2, which also shows crude and sex-specific mortality rates and mortality rate ratios comparing males with females.

**Table 2:** Neonatal verbal autopsies (VA) included in the study

				Neonatal mortality rate (per 1000 live births)			
Study site	Live births included	Stillbirths included (proportion of all deaths)	Neonatal deaths included (proportion of all deaths)	Total	Male	Female	Crude neonatal mortality rate ratio (male: female) (95% CI)
Ekjut, rural India	8819	270 (34%)	518 (66%)	59	68	50	1.4 (1.1 to 1.6)
SNEHA, urban India	10 029	80 (48%)	87 (52%)	9	10	7	1.3 (0.8 to 2.1)
Dhanusha, Nepal	15 299*	463 (47%)	528 (53%)	35	39	30	1.3 (1.1 to 1.5)
Makwanpur, Nepal	6735	146 (42%)	203 (58%)	30	36	24	1.5 (1.1 to 2.0)
PCP, rural Bangladesh	42 241	1361 (51%)	1324 (49%)	31	36	27	1.3 (1.2 to 1.5)
MaiMwana, Malawi	15 258	324 (46%)	382 (54%)	25	29	21	1.4 (1.1 to 1.7)
MaiKhanda, Malawi	22 563*	612 (46%)	730 (54%)	32	35	30	1.2 (1.0 to 1.4)

*Estimated from available data.

PCP, Perinatal Care Project; SNEHA, Society for Nutrition, Education and Health Action.

**Figure 1:**
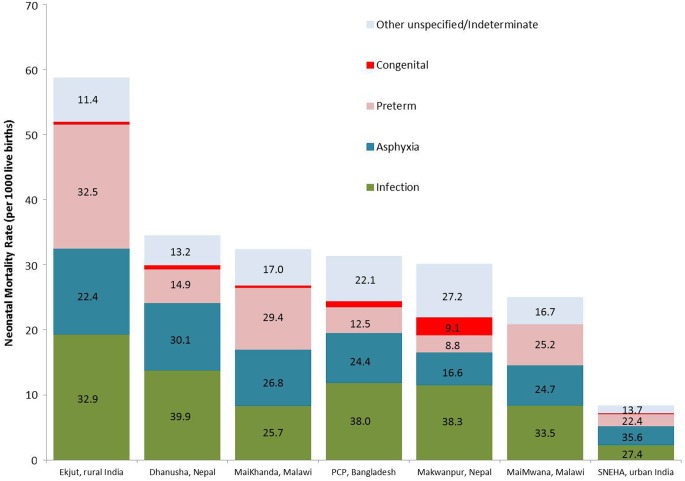
Total and cause-specific neonatal mortality rates by study site ordered left to right by decreasing neonatal mortality rate. Superimposed numbers represent cause-specific mortality fractions (CSMFs) (%) for each site. PCP, Perinatal Care Project; SNEHA, Society for Nutrition, Education and Health Action.

Table 3 shows sex-specific and cause-specific neonatal mortality fractions for all InterVA-derived cause categories. Infectious causes—diarrhoeal disease, meningitis and encephalitis, pneumonia and sepsis—were consolidated into a broad cause category of infection, and the categories ‘other and unspecified’ and ‘indeterminate’ were consolidated to facilitate presentation of cause-specific rates and overall fractions in figure 1.

**Table 3:** Cause and sex-specific neonatal mortality fractions and corresponding International Classification of Diseases V.10 (ICD-10) code

	Ekjut, rural India			SNEHA, urban India			Dhanusha, Nepal			Makwanpur, Nepal			PCP, Bangladesh			MaiMwana, Malawi			MaiKhanda, Malawi		
Cause and corresponding ICD classification	M	F	Total	M	F	Total	M	F	Total	M	F	Total	M	F	Total	M	F	Total	M	F	Total
10.01 prematurity	31.3	34.2	32.5	16.8	30.8	22.4	16.4	12.9	14.9	7.0	11.7	8.8	12.1	13.0	12.5	22.3	28.9	25.2	26.4	32.7	29.4
01.04 diarrhoeal diseases		0.5	0.2										0.2	0.2	0.2						
01.07 meningitis and encephalitis	5.0	4.0	4.6	1.1	2.4	1.6	1.0	1.3	1.1	1.2	1.2	1.2	2.1	1.6	1.9	1.2	2.4	1.7	2.7	1.2	2.0
10.03 neonatal pneumonia	19.6	19.0	19.4	22.8	20.6	21.9	24.0	25.6	24.6	35.7	21.0	29.9	29.9	29.9	29.9	26.2	19.6	23.3	13.9	14.9	14.3
10.04 neonatal sepsis	9.0	8.5	8.8	5.6	1.3	3.9	12.7	16.3	14.2	6.5	8.4	7.2	5.9	6.4	6.1	8.1	8.9	8.5	9.7	9.1	9.4
*Infections (01.04+01.07+10.03+10.04)*	*33*.*6*	*32*.*1*	*32*.*9*	*29*.*5*	*24*.*3*	*27*.*4*	*37*.*6*	*43*.*1*	*39*.*9*	*43*.*4*	*30*.*5*	*38*.*3*	*38*.*0*	*38*.*1*	*38*.*0*	*35*.*5*	*30*.*9*	*33*.*5*	*26*.*3*	*25*.*2*	*25*.*7*
10.02 birth asphyxia	22.9	21.6	22.4	39.8	29.4	35.6	31.0	29.0	30.1	15.6	18.1	16.6	23.7	25.5	24.4	24.4	25.0	24.7	29.3	24.1	26.8
10.06 congenital malformation	0.7	0.9	0.8		2.3	0.9	1.3	2.7	1.9	10.1	7.6	9.1	3.2	2.6	3.0				0.5	1.7	1.1
10.99 other and unspecified	1.2	1.3	1.2	1.7	2.7	2.1	6.2	4.4	5.4	8.2	7.6	7.9	7.6	6.3	7.1	8.2	5.1	6.89	8.5	8.6	8.6
99 indeterminate	10.4	9.9	10.2	12.4	10.4	11.6	7.7	7.9	7.8	15.9	24.4	19.2	15.4	14.6	15.1	9.6	10.0	9.8	9.0	7.8	8.4

ICD, International Classification of Diseases; PCP, Perinatal Care Project; SNEHA, Society for Nutrition, Education and Health Action.

Crude sex-specific neonatal mortality rates and rate ratios (table 2) show that males had 20%–50% higher mortality than females, with rate ratios (95% CIs) ranging from 1.2 (1.0–1.3) in MaiKhanda to 1.5 (1.1–2.0) in Makwanpur. In most settings, there were no obvious important differences between cause distributions by sex (table 3). However, at SNEHA in urban India and, to a lesser extent, Makwanpur in Nepal, a larger proportion of female deaths were attributed to prematurity.

**Figure 2:**
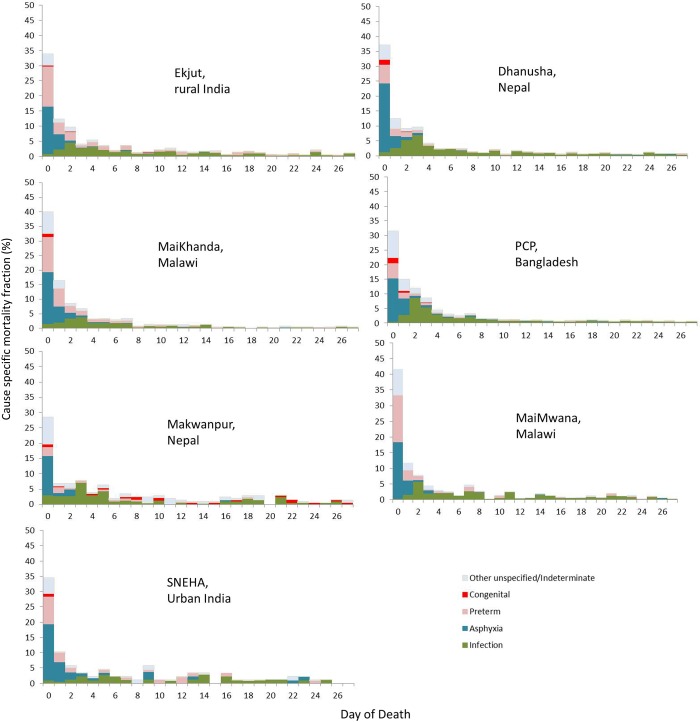
Cause-specific mortality fractions (CSMFs) by day of death and study site ordered left to right by decreasing neonatal mortality rate. PCP, Perinatal Care Project; SNEHA, Society for Nutrition, Education and Health Action.

Figure 2 shows CSMFs by day of death. Across all Asian sites, approximately one-third of neonatal deaths occurred on the first day of life. This proportion was greater than 40% in both Malawian sites, and, in all settings, the first week of life accounted for most neonatal deaths, ranging from 63% in Makwanpur (Nepal) and SNEHA (India) to 82% in MaiKhanda (Malawi).

## Discussion

Using prospective data from seven community-based surveillance systems, we applied an automated method of VA interpretation to provide a uniquely standardised summary and comparison of neonatal mortality across four low-income and middle-income settings. Across all settings, and particularly in rural areas, neonatal mortality rates were unacceptably high and driven by prematurity, birth asphyxia (intrapartum-related deaths) and infections, which were particularly important in the rural Asian sites. The first days of life remain the most risky, with more than one-third of deaths occurring on the first day, half in the first 2 days and three-quarters in the first week.

### Gender disparities

Male gender disadvantage was observed in all study sites, with boys being up to 50% more likely to die than girls. While our observation could be a consequence of methodological bias, whereby social desirability may lead to an over-reporting for male offspring, a neonatal mortality ratio of males to females of at least 1.2 has been reported previously.^37^ ^38^ Recent research has also shown that boys are more likely to be born prematurely and have a higher incidence of infections and encephalopathy.^39–41^ With the exception of Dhanusha (Nepal) and PCP (Bangladesh), sex differentials in CSMFs in table 3 suggested more infections in males, although prematurity appeared to act in the opposite direction, and was responsible for a higher proportion of female deaths. Similar findings for late neonatal deaths have been observed in other studies from Nepal^38^ and India,^42^ and may reflect overarching gender preference or preferential care-seeking as well as interactions between ethnicity, sex and the presence of prior siblings. The extent to which observed sex differentials reflect biological phenomena or are a consequence of modifiable socioeconomic factors demands further investigation.

### Differences between countries

There were important differences in overall mortality and cause distributions between settings. PCP (Bangladesh) and the sites in Malawi and Nepal had similar overall neonatal mortality rates of 25–35 deaths per 1000 live births. However, the proportion of deaths attributed to prematurity, birth asphyxia and infections varied considerably between settings. In the Bangladesh site and both Nepal sites, the burden of the combined infectious causes of death accounted for almost two-fifths of the total mortality burden, whereas in Malawi, the burden ranged from 26% to 34%, and a greater proportion of prematurity-related mortality was observed. This is perhaps unsurprising given that Malawi is reported to have the highest prematurity rate in the world, at 18%.^43^

### Differences within countries

Ekjut, in rural India, had the highest neonatal mortality rate of 59 deaths per 1000 live births, almost seven times greater than SNEHA in urban Mumbai, reflecting huge subnational variation. Infections and prematurity each accounted for around one-third of neonatal deaths in Ekjut. While infections and prematurity remained in the top three causes in Mumbai, more than one-third of deaths were attributed to asphyxia, making it the leading cause of death. This is in agreement with a neonatal VA study from the same setting, which identified one-third of asphyxia deaths as being associated with obstetric complications.^44^ Observed differences between rural and urban India are likely to reflect documented shifts in cause patterns as overall mortality rates fall, largely driven by decreases in the rates of death from infections, particularly pneumonia.^45^

Within-country differences were also observed in Malawi and Nepal, but are not easily explained by underlying mortality rates. In Malawi, data from MaiMwana suggest that approximately one-third of neonatal deaths were attributable to neonatal pneumonia/sepsis, approximately 8% greater than the burden identified at the MaiKhanda site, where slightly higher burdens of prematurity and asphyxia were observed. Both MaiMwana and MaiKhanda represent rural Malawian populations, but at slightly different times and within a period of rapid increase in institutional deliveries.^36^ More births occurred in facilities in MaiKhanda than in MaiMwana (table 1). Given that home deliveries are associated with less hygienic practices and higher rates of infectious causes of neonatal death,^46^ the higher proportion of institutional delivery in MaiKhanda may partly explain the differences in cause-specific mortality. A similar scenario may also explain differences observed between Makwanpur (2% facility deliveries) and Dhanusha (26% facility deliveries) in Nepal. However, the different time periods for data collection and contextual differences between the plains of Dhanusha and the hills of Makwanpur may also be important. Finally, the substantial difference in the proportion of deaths with insufficient VA data to attribute a specified cause (categorised as ‘indeterminate’ and ‘other unspecified’ in table 3) suggests that observed differences in cause distribution may also relate to underlying differences in VA data completeness.

### Study strengths and limitations

The prospective birth and death surveillance systems, high follow-up rate for completion of VAs, data quality procedures and large sample size are the strengths of the study. There remains a potential bias if certain neonatal deaths were systematically unsuccessfully followed up for VA, but we do not expect this given that the sum of the VA-derived CSMRs closely approximated total mortality rates derived from surveillance in the same areas.^17–23^ The use of InterVA is also a strength in that it allows identification of multiple causes of death per case with quantified degrees of certainty, provides confidence that observed differences are not a consequence of differing methods of assigning causes and has been shown to compare well with available reference standards for cause of death in a range of settings,^47^ including perinatal death classifications.^48^ Indeed, the CSMFs identified by InterVA for Dhanusha and SNEHA were similar to those derived by physician review.^44^ ^49^ Cause distributions were also similar between InterVA and physician interpretation of neonatal VA data from Ekjut and PCP (unpublished data). However, physicians at PCP identified asphyxia as the leading cause of death, and Ekjut physicians attributed a substantial number of neonatal deaths to hypothermia, which is not one of the causes included in the 2012 WHO VA standards and, therefore, in InterVA, which may have largely classified such deaths as ‘indeterminate’.

Several methodological limitations might have influenced the cause distributions observed. The specific questionnaires used at each site, though similar, were not exactly the same, and the period between neonatal death and VA interview differed. In addition to the potential biases inherent in survey methods and maternal reporting,^50^ ^51^ these differences may have influenced VA respondents’ ability to recognise, recall and report signs and symptoms. They may also partly explain the differing mean number of non-missing indicators reported per case in each setting, ranging from 12 in MaiKhanda to 17 in Bangladesh. Similarly, it is possible that the processing of common questions into site-specific translations for InterVA may have led to subtle differences in meaning and comprehension. Although there is no empirical evidence on the effect of such factors on VA-derived causes of death, and InterVA was designed with the reality of different VA data collection tools in mind (eg, only affirmative reports of the presence of indicators influence cause probabilities), the possibility that differing cause distributions are driven by differing data capture methods cannot be ruled out.

### Comparisons with other studies

In general, our analysis supports current modelled characterisations of neonatal mortality at an aggregate level.^45^ Our cause-distribution findings are also similar in magnitude and rank order to survey results reported elsewhere for comparable settings,^44^ ^49^ ^52^ ^53^ and are broadly similar to descriptions of neonatal mortality from 18 INDEPTH health and demographic surveillance sites using similar methods.^54^ There are some notable exceptions. Chowdhury *et al*^55^ identified birth asphyxia as the leading cause among 365 neonatal deaths in Matlab, rural Bangladesh, accounting for 45% of the total burden, whereas infection-related causes accounted for less than 20% of deaths. This contrasts with our findings from Bangladesh, which attributed approximately one-quarter of deaths to asphyxia and a much higher proportion to infections. Conversely, comparisons of our findings with public domain INDEPTH data^56^ for 1619 and 207 neonatal deaths from Bangladesh and India, respectively, identify higher CSMFs of asphyxia and lower proportions of other unspecified/indeterminate causes in our data. While it is possible that such differences reflect varying epidemiology and rates of skilled attendance at birth between different areas, it is also conceivable that they are an artefact of differing VA survey methods. The Matlab study, for example, had a relatively small sample size, and VA interpretation was by a panel of physicians required to reach consensus on a single cause of death for each case.^55^ Neonatal causes of death can be difficult to attribute with certainty, depending on clinical factors and issues of definition; a recent analysis showed a fivefold difference in the proportion of deaths attributed to prematurity depending on definition used.^57^ As such, identifying a single direct cause of death from VA may be unrealistic and masks the uncertainty that may have been evident in the original physicians’ consideration of individual cases. Our method, and that used by the INDEPTH group, of calculating population-level CSMFs and splitting deaths between multiple causes is arguably a more epidemiological approach that shifts the focus from individuals to populations.^58^ This fundamental methodological difference may explain the lower overall proportion of asphyxia deaths observed in the INDEPTH datasets as well as ours compared with the study by Chowdhury *et al*.^55^ Given that both PCP and the INDEPTH data were interpreted using InterVA V.4.02 however, differences between these datasets must be due to other reasons, possibly underlying differences in the time and location of the surveys, or differences in VA data capture.

### Programmatic implications

At all study sites, stillbirths accounted for a large proportion of deaths in the extended perinatal period. The estimates in our data ranged from 34% in Ekjut, India, to 51% in PCP, Bangladesh. All methods used to distinguish stillbirths from early neonatal deaths are open to misclassification bias, influenced by sociocultural factors and limited assessment of vital signs or attempts at resuscitation in apparently lifeless newborns, particularly after home birth.^59^ Misclassification bias notwithstanding, the burden of stillbirths remains high and is likely to reflect inadequacies in skilled attendance at birth and the availability of emergency obstetric care and caesarean sections.

The neonatal cause-of-death distributions described here have important programmatic implications. High levels of infection and prematurity-related deaths imply that essentials of hygienic delivery and newborn care, including thermal care, remain priorities. Preventative strategies that target maternal health, recognising the importance of nutrition and vector-borne diseases such as malaria, a major cause of anaemia in malaria endemic regions, must also be prioritised. At the same time, the major burden of asphyxia mortality demands a focus on the intrapartum period, with improved capacity within facilities to manage obstetric complications and awareness of the importance of skilled attendance at birth among communities and expectant mothers. Given the high rates of home births, intervention strategies must think beyond the biomedical aspects of mortality described by VA results and consider the distal, social determinants of mortality and the three delays model.^60^ In this regard, there is a clear need to focus on community interventions for newborn health that also encompass the health of the mother and the continuum of preconception, pregnancy and the intrapartum and postpartum periods, and which promote hygienic practices, thermal care, the recognition of danger signs and appropriate care-seeking. There is a growing body of evidence suggesting that cost-effective community interventions achieving impact on neonatal mortality should be scaled up as a matter of urgency.^24^ ^61^ ^62^

## Conclusion

The application of simple mortality surveillance and VA methods in localised, low-income and middle-income settings reveals important regional and subnational variation in the epidemiology of neonatal mortality that may be masked by aggregate or modelled estimates. Although population health survey and VA approaches are likely to be imperfect compared with complete vital registration and medical cause of death certification, methodological advances enable the generation of timely, low-cost and consistent cause-specific mortality estimates necessary to direct local policy in settings with little or no existing data. The wider application of these methods beyond research settings to better inform and strengthen health systems is a global priority. However, it is not for lack of data that the burden of neonatal mortality remains so high; efforts must focus on implementing proven interventions to protect the three million newborns who die every year.

## Supplementary Material

Web supplement
