# Supplementary material for: Cause-specific neonatal mortality: analysis of 3772 neonatal deaths in Nepal, Bangladesh, Malawi and India
Source: Arch Dis Child Fetal Neonatal Ed. 2015 May 13;100(5):F439–47. doi: 10.1136/archdischild-2014-307636 (PMC4552925; doi:10.1136/archdischild-2014-307636)
Supplement: Web supplement [file fetalneonatal-2014-307636-s1.pdf]

Table S1 113 verbal autopsy indicators applicable to neonatal deaths in InterVA and their availability (✓) in the peri/neonatal VA questionnaire used in each site.

VA Indicator	Dhanusha	MaiMwana	Ekjut	PCP	Maikhanda	Makwanpur	SNEHA
Was the deceased male?	✓	✓	✓	✓	✓	✓	✓
Was the deceased female?	✓	✓	✓	✓	✓	✓	✓
Was (s)he aged 28 days or less at death?	✓	✓	✓	✓	✓	✓	✓
Did the baby die within 24 hours of birth?	✓	✓	✓	✓	✓	✓	✓
Did baby die more than 24 hours after but less than 48 hours from birth?	✓	✓	✓	✓	✓	✓	✓
Did baby die more than 48 hours from birth but within the first week of life?	✓	✓	✓	✓	✓	✓	✓
Did baby die after first week, but within first 28 days?	✓	✓	✓	✓	✓	✓	✓
Was there any diagnosis of Measles?							
Did (s)he die during the wet season?	✓	✓	✓	✓	✓	✓	✓
Did (s)he die during the dry season?	✓	✓	✓	✓	✓	✓	✓
Did (s)he have a fever?	✓	✓	✓	✓	✓	✓	✓
Did (s)he have fever for less than 2 weeks before death?	✓	✓	✓	✓	✓	✓	✓
Did (s)he have fever for 2 weeks or more before death?	✓	✓	✓	✓	✓	✓	✓
Did (s)he have a cough?			✓	✓		✓	
Did (s)he have a cough for less than 3 weeks before death?						✓	
Did (s)he make a whooping sound when coughing?							
Did (s)he have any breathing problem?	✓	✓	✓	✓	✓	✓	✓
Did (s)he have fast breathing?	✓	✓	✓	✓	✓	✓	✓
Did (s)he have fast breathing for less than 2 weeks?		✓			✓	✓	✓
Did (s)he have breathlessness?							✓
Did (s)he have breathlessness for less than 2 weeks?							✓
Did you see the lower chest wall/ribs being pulled in as the child breathed?	✓	✓	✓	✓	✓		✓
Did (s)he have noisy breathing (grunting or wheezing)?	✓	✓		✓		✓	✓
Did (s)he have diarrhoea?	✓	✓	✓	✓	✓	✓	✓
Did (s)he have diarrhoea for less than 2 weeks?	✓	✓	✓	✓	✓	✓	✓
Did (s)he have diarrhoea for 2 to 4 weeks?	✓	✓	✓	✓	✓	✓	✓
At any time during the final illness was there blood in the stools?	✓	✓	✓	✓	✓	✓	
Did (s)he vomit?	✓	✓	✓	✓	✓	✓	✓

Did (s)he vomit "coffee grounds" or bright red/blood?							
Did (s)he have any abdominal problem?			✓	✓		✓	✓
Did (s)he have a more than usually protruding abdomen?				✓			✓
Did (s)he have a more than usually protruding abdomen for less than 2 weeks?							
Did (s)he have convulsions?	✓	✓	✓	✓	✓	✓	✓
Did the convulsions last for less than 10 minutes?							
Did the convulsions last for 10 minutes or more?							
Did (s)he have any skin problems?	✓		✓	✓	✓	✓	✓
Did (s)he have any ulcers, abscess or sores anywhere except on the feet?							
Did (s)he have any ulcers, abscess or sores on the feet that were not also on other parts of the body?							
During the illness that led to death, did (s)he have any skin rash?	✓	✓			✓		
Did (s)he have the skin rash for less than 1 week?							
Did (s)he have the skin rash for 1 week or more?							
Did (s)he have measles rash?							
Did (s)he have yellow discoloration of the eyes?	✓	✓			✓	✓	✓
Did (s)he look pale (thinning/lack of blood) or have pale palms, eyes or nail beds?							
Was the child part of a multiple birth?	✓	✓	✓	✓	✓	✓	✓
Was the child born in a complicated delivery?	✓	✓	✓	✓	✓	✓	✓
Was the baby born 24 hours or more after the water broke?	✓			✓		✓	✓
Did the baby stop moving in the womb before labour started?	✓	✓	✓	✓			
Was baby born in a normal vaginal delivery?	✓	✓	✓	✓	✓	✓	✓
Was baby born with forceps/vacuum?	✓	✓	✓	✓	✓	✓	✓
Was baby delivered by Caesarean section?	✓	✓	✓	✓	✓	✓	✓
Did the baby's bottom, feet, arm or hand come into the vagina before its head?	✓	✓	✓	✓	✓	✓	✓
Was the baby of abnormal size?	✓	✓	✓	✓	✓	✓	✓
Was the baby smaller than normal, weighing under 2.5 kg?	✓	✓	✓	✓	✓	✓	✓
Was the baby larger than normal, weighing over 4.5 kg?	✓	✓	✓	✓	✓	✓	✓

Had the pregnancy lasted less than 34 weeks when the baby was born?	✓	✓	✓	✓	✓	✓	✓
Had the pregnancy lasted between 34 and 37 weeks when the baby was born?	✓	✓	✓	✓	✓	✓	✓
Had the pregnancy lasted more than 37 weeks when the baby was born?	✓	✓	✓	✓	✓	✓	✓
Was the umbilical cord wrapped several times (more than once) around the neck of the child at birth?							✓
Did the baby/the child have any noticeable malformation?	✓	✓	✓	✓	✓	✓	✓
Did the baby/the child have a swelling/defect on the back?	✓	✓	✓	✓	✓		✓
Did the baby/the child have a very large head?							
Did the baby/the child have a very small head?	✓	✓	✓	✓	✓	✓	✓
Was the baby blue in colour at birth?			✓	✓		✓	
Did the baby cry after birth, even if only a little bit?	✓	✓		✓		✓	✓
Did the baby breathe after birth, even a little?	✓	✓	✓	✓	✓	✓	✓
Was the baby given assistance to breathe at birth?	✓	✓	✓	✓	✓	✓	✓
If the baby did not cry or breathe, was it born dead?	✓	✓	✓	✓	✓	✓	✓
Was the dead baby macerated, that is, showed signs of decay?	✓	✓	✓	✓	✓	✓	✓
Was the baby able to suckle or bottle-feed within the first 24 hours after birth?	✓	✓	✓	✓	✓	✓	✓
Did the baby stop suckling or bottle feeding 3 days after birth?	✓	✓	✓	✓	✓		✓
Did the baby have convulsions starting within the first day of life?	✓	✓	✓	✓	✓	✓	✓
Did the baby have convulsions starting on the second day or later after birth?	✓	✓	✓	✓	✓	✓	✓
Did the baby's body become stiff, with the back arched backwards?	✓	✓	✓	✓	✓	✓	✓
Did the child have a bulging or raised fontanelle?	✓	✓	✓	✓	✓	✓	✓
Did the child have a sunken fontanelle?	✓	✓	✓	✓	✓	✓	
Did the baby become unresponsive or unconscious soon after birth (within less than 24 hours)?	✓	✓	✓	✓			✓
Did the baby become unresponsive or unconscious more than 1 day after birth?		✓	✓	✓			✓
Did the baby become cold to the touch before it died?	✓	✓	✓	✓	✓	✓	✓

Did the baby have redness or discharge from the umbilical cord stump?	✓	✓	✓	✓	✓	✓	✓
Did the baby have yellow palms or soles?	✓	✓	✓	✓			✓
Did the mother NOT receive tetanus toxoid (TT) vaccine?			✓	✓			
Was this baby born from the mother's first pregnancy?			✓	✓		✓	✓
Did the mother have 4 or more births before this baby was born?			✓	✓			✓
During pregnancy, did the baby's mother suffer from high blood pressure?			✓			✓	✓
Did the baby's mother have foul smelling vaginal discharge during pregnancy or after delivery?			✓	✓		✓	
During the last 3 months of pregnancy, did the baby's mother suffer from convulsions?			✓	✓		✓	✓
During the last 3 months of pregnancy did the baby's mother suffer from blurred vision?			✓	✓		✓	
Did the baby's mother have vaginal bleeding during the last 3 months of pregnancy but before labour started?			✓	✓		✓	✓
Was the child born in a health facility?	✓	✓	✓	✓	✓	✓	✓
Was the child born at home?	✓	✓	✓	✓	✓	✓	✓
Was the child born somewhere else, e.g. on the way to a facility?	✓	✓		✓	✓	✓	✓
Did the mother receive professional assistance during the delivery?	✓	✓	✓	✓	✓	✓	✓
Did (s)he suffer from any injury or accident that led to her/his death?	✓		✓	✓	✓	✓	✓
Was it a road traffic accident?	✓						
Was (s)he Injured in a non-road transport accident?							
Was (s)he Injured in a fall?	✓						
Did (s)he die of drowning?	✓						
Did (s)he suffer from burns?	✓						
Did (s)he suffer from any plant/ animal/insect bite or sting that led to her/his death?	✓						
Was (s)he injured by a force of nature?							
Was there any poisoning?	✓						
Was (s)he subject to violence/assault?							
Was the injury intentionally inflicted by someone else?							
Was (s)he adequately vaccinated?	✓	✓			✓		
Did (s)he receive any treatment for the illness that led to death?	✓						✓

Did (s)he receive oral rehydration salts?							
Did (s)he receive (or need) intravenous fluids (drip) treatment?							✓
Did (s)he receive (or need) a blood transfusion?							
Did (s)he receive (or need) injectable antibiotics?							
Did (s)he have (or need) an operation for the illness?							
Did (s)he have the operation within 1 month before death?							
Was (s)he discharged from hospital very ill?							
